# *Schistosoma mansoni* and soil transmtted helminths in olive baboons and potential zoonosis

**DOI:** 10.1002/vms3.495

**Published:** 2021-05-04

**Authors:** Maloba Fredrick, Mwangi Danson, Kagira John, Kivai Stanislaus, Ndeereh David, Ngotho Maina, Gicheru Michael, Mbaruk Suleiman, Akinyi Mercy

**Affiliations:** ^1^ Department of Zoological Sciences Kenyatta University Nairobi Kenya; ^2^ Department of Conservation Biology Institute of Primate Research Nairobi Kenya; ^3^ Jommo Kenyatta University of Agriculture and Technology Nairobi Kenya; ^4^ Kenya Wildlife Service Nairobi Kenya; ^5^ Mount Kenya University Thika Kenya; ^6^ Department of Biology Duke University Durham NC USA

**Keywords:** baboon, Schistosomiasis, Zoonoses

## Abstract

Zoonotic pathogens are among the most important causes of ill health all over the world. The presence of these pathogens in free ranging baboons may have significant implications for humans. In Kenya, the encroachment of wildlife habitats has led to increased interaction between humans and wildlife especially non‐human primates. The current study therefore aimed at investigating any possible zoonotic gastrointestinal helminths of olive baboons (*Papio anubis*) at the human–wildlife interface in two park borders and a ranch in Kenya, namely, Tsavo West National Park, Tana River Primate Reserve and Mutara Ranch, Laikipia, Kenya. One hundred and forty‐seven baboons were used in the study. They were trapped in the wild, sampled for stool marked and then released back to the wild. Gastrointestinal (GIT) helminths identified were *Strongyloides, Oesophagostomum, Enterobius spp* and *Trichuris Trichiura* from all the three sites while *Schistosoma mansoni* was only detected from Tsavo baboons and with very low incidence (2.1%). The prevalence of these parasites varied among the sites but significant difference in prevalence was only noted in *Strongyloides* and *Oesophagostomum* (*p* < 0.05) among the three sites. This therefore implies that even with control measures instituted on the human population, baboons will always be a source of zoonotic GIT helminths especially *S. mansoni* even if the incidence are low. There is need to put in place measures aiming to reduce their interactions with humans and also try to control these infections in the baboons.

## Graphical abstract

Baboons harbor Schistosoma mansoni which is infectious to humans. Humans can be infected through sharing of water resources, eating left overs since their fecal waste contaminate water and food. Control of the schistosomiasis in human population alone will still be a challenge if possible zoonotic sources are not looked at.

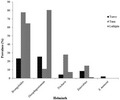

## 1 INTRODUCTION

Schistosomiasis and soil transmitted helminths (STHs) are among the Neglected Tropical Diseases (NTDs) that can be controlled by chemotherapy. A report in 2013 indicated that more than 290 million people worldwide were estimated to be infected with schistosomiasis, about 600–780 million being at risk of infection, with morbidity due to these infections resulting to an estimated 2.8 million disability adjusted life years (DALYs) (Global burden of disease, 2013). There have been accelerated efforts by WHO to eliminate these NTDs through mass deworming (WHO, 2011). Despite the efforts, schistosomiasis still remains a challenge with hot spots still being reported in various parts of the country (Sang et al., 2014). Both schistosomiasis and soil transmitted helminths are also major causes of anemia in pregnant women in addition to schistosomiasis causing female genital schistosomiasis (Salam et al., 2019). With the much effort put on the control of the helminth in humans, there still remains a challenge in controlling the zoonotic aspect. There are reports of human interaction to wildlife which most of the times lead to human wildlife conflict (HWC) (Makena et al., 2019, Mwangi et al., 2016) as well as zoonoses transmission (Fredrick et al., 2019). Non‐human primates have been shown to harbor most of the zoonoses which could be probably due to their high physiological closeness to humans (Cox et al., 2013; Rogers & Gibbs, 2014). There are reports of zoonotic schistosomiasis especially *S. mansoni* in non‐human primates hence these animals are likely to serve as reservoirs for the parasite (Richards et al., 2019; Standley et al., 2012). Other zoonotic diseases in non‐human primates include viral infections such as herpes B encephalitis, simian foamy virus (SFV) and simian T‐cell leukemia virus‐1 **(**STLV‐1), bacterial (Conly & Johnston, 2008), helminths (Mafuyai et al., 2013) and protozoans (Munene et al., 1998) as well as fungal infections. Among the primates with the highest interaction are the baboons and vervet monkeys as they cause crop raids and livestock predation in search for food (Mwangi et al., 2016). During these attacks, they contaminate water resources and the human environment with their fecal and urine waste (Travis et al., 2006). In addition, due to the poverty caused by the destruction of crops and livestock, humans mostly feed on leftovers from the raids which are also likely to be contaminated by the animal's saliva. The current study therefore sought to find out the gastrointestinal helminths in the baboons at the human wildlife borders.

## 2 MATERIALS AND METHODS

### 2.1 Study sites

**FIGURE 1:**
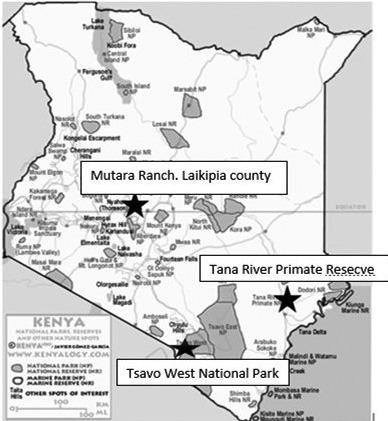
Map indicating three study sites for baboon sampling. Asteric showing the locations (Research gate, 2015). The sites were selected due to the possible high interaction of baboons with humans

The study areas included Tana River Primate National Reserve (TRPNR), Tsavo West National Park (TWNP) and Mutara ranch in Laikipia. In these areas, humans closely interact with baboons (Figure 1). Tana River Primate National Reserve is a non‐human primate (NHPs) conservation site where humans and NHPs share the habitat and a common water source‐ River Tana. The forest is a good habitat for NHPs while humans farm along the river bank and also use the water for domestic activities and watering livestock. Tana River flows in the Lower Tana River gallery forest which is one of the rare yet complex habitats in eastern Africa. The lower flood plain of the Tana River, the largest river in Kenya contains a unique community of riverine forest vegetation in scattered patches of various sizes. The forest is a relic of a previously more continuous rainforest that extended from the Congo River Basin to the east coast of Africa during the Pleistocene era. Later, prolonged droughts led to the shrinkage and isolation of east African evergreen forests, leaving them confined to moist highland and riverine areas (Africa‐horizons, 2020). The forest is unique because it supports a high diversity of plant and animal species. It is about 100 km upstream from the Tana delta, between latitudes 2°15^′^ and 1°50′ south (Tana River County, 2015).

Tsavo West National Park was selected because there are human habitations around the park that practise both livestock and crop cultivation and baboons are also ubiquitous in the park. The site consists mainly of semi‐arid plains, granite outcrops and ancient lava fields. For most of the year, Tsavo is dry and dusty. Generally, the weather in Tsavo is warm and dry, with temperatures ranging from 20to40°C and rainfall from 200to700 mm per year. There are two rainy seasons; the long rains are generally from March through to May when the weather is hot and humid while the short rains come in the warm months of October to December (Fallingrain, 2015). The study areas in this site were the northern parts—Nthongoni location with targeted villages including Matangini, Yumbuni, Mangelete, Nthongoni, Kongo and California which border the park.

The third site was Mutara ranch which is a government owned beef cattle ranch located in Laikipia County. The climate is predominantly semi‐arid and the ranch is a habitat for feral baboons among other animals (AWF, 2007). The baboons share the resources with the cattle and humans hence the higher risk of zoonotic parasite transmission. Humans also live around the ranch and there are tourist hotels found near the ranch. Baboons therefore move around in neighbouring farms and hotels scavenging for food hence interacting with humans and this is what necessitated its selection.

### 2.2 Field capture and release of baboons

The baboons were habituated then trapped before being anaesthetized using Xylazine/Ketamine mixture at the ratio of 20:1 respectively at a dose rate of 10 mg/kg (0.5 mg/kg and of Ketamine and 9.5 mg/kg of Xylazine). Once under deep sedation, the animals were given identification marks to avoid resampling upon re‐capture. Data on location, species, sex, age were recorded. The animals were then observed for recovery before being released back to the wild. For those that took longer than 30 min, the anesthesia was reversed using Atipamazole hydrochloride (Atepam®, Cipla ltd, India) at 0.5‐mg/kg body weight to hasten the recovery.

### 2.3 Fecal sample collection and analysis

Fecal sample was collected directly from the rectum of the anaesthetized animal. This was done by ensuring the finger nails were all short to avoid rectal injury. The hands were then gloved then dipped in clean water to reduce friction when inserted in the animal rectum. For juveniles, only one finger would be inserted while for adults a maximum of two fingers were inserted in the rectum. About 5grams of stool was scooped. This is the first time this procedure was carried out otherwise most of the direct samples are usually swabs which would not have been sufficient for parasitological analysis (Artim et al., 2019; Lu et al., 2020). The direct rectal collection was also done since stool once passed out is usually prone to environmental contamination (Davoust et al., 2018). The sample was then placed in 50‐ml tubes with 10% formalin for preservation. The fecal sample was later used for both identification of any eggs recovered and their quantification into eggs per gram (EPG) by sedimentation technique as described by Gillespie (2006). The fecal sample was thoroughly homogenized with a stirring stick then 2 g of the sample weighed out into a plastic container. Twelve milliliters of water was added to the sample then stirred thoroughly to form a thin brown slurry. To remove the large debris, the sample was swirled to suspend the sediment and the slurry poured through a strainer measuring 20‐cm diameter and 5cm high with a pore size of 425µm into another fecal sample jar. The filtrate was swirled to suspend the sample and then poured into a 15‐ml conical tube. If the sample volume was less than 14ml the tube was filled to the 14‐ml mark with water. The tubes were then capped and the samples centrifuged at 1,500 rpm for 10 min. The supernatant was then poured off and 750 µl of tap water added to the sample sediment followed by 500 µl of a concentrated sugar solution (prepared by dissolving 454 g of sugar in 355 ml of hot water). The mixture was thoroughly mixed then two drops of the sediment placed on a slide and examined under a microscope at 10X. The slide was then scanned for the presence of eggs. For quantification of eggs, counting was done systematically from one corner of the slide moving upwards and downwards in a line, then next until finished. The number of each type of egg was noted. For EPG, the number was multiplied by 70.

## 3 RESULTS

### 3.1 Helminth prevalence

**FIGURE 2:**
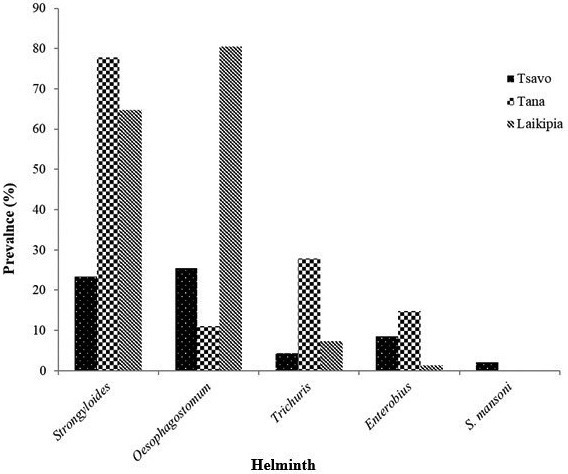
Prevalence rates of gastrointestinal tract helminths among baboons from the study sites. *Schistosoma mansoni* which is a human trematode was present though a low prevalence in addition to other zoonotic helminths

A total of 107 baboons were sampled; 47 from Tsavo and 60 from Tana River. Point prevalence rates of gastrointestinal tract (GIT) helminths was determined for three study sites. Tana River Primate Reserve baboons had the higher prevalence of all the parasites *Strongyloides* (77.8%, 23.3%)*, Trichuris* (27.8%, 4.3%), *Enterobius* (14.8%, 8.5%) except for *Oesophagostomum* (11.1%, 25.5%) and *S. mansoni* (2.1%, 0%) in Tana and Tsavo respectively (Figure 1). ANOVA was performed in comparing prevalence rates in the study sites. With regard to parasites, *Oesophagostomum* had the highest infection rates followed by *Strongyloides* then *Trichuris*, *Enterobius* and lastly *S. mansoni*. There was a significant difference in prevalence of *Strongyloides* between the sites (*p* <0.05), with Tana River having higher prevalence (77.8%) as compared to Tsavo (23.4%). *Oesophagostomum* prevalence was also significantly different between the three study sites (*p* <0.05) (Tsavo 25.5%, Tana 11.1%). *Trichuris* (*p* <0.05) was equally significantly different between the sites with Tana River having a higher prevalence (27.5%) compared to Tsavo (4.3%). *Enterobius* was also significantly different between the study sites with Tana having the higher prevalence (14.8%) followed by Tsavo (8.5%). There was no significant difference in the prevalence of *S. mansoni* (*p* >0.05) among the sites with the parasite only being reported in Tsavo with a very low prevalence of 2.1% (Figure 2).

### 3.2 Helminth infection intensity

**FIGURE 3:**
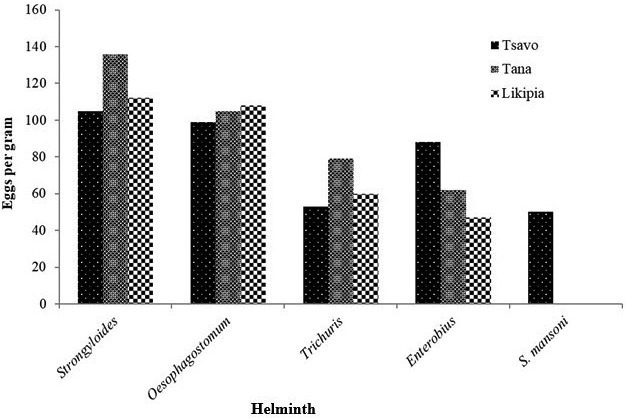
Mean egg per gram counts plot for helminths from the three study sites. *Schistosoma mansoni* reported the lowest intensity infection which would indicate that the baboon could be managing the infections hence acting as carriers

Mean eggs per gram (EPG) of feces was calculated for each site. In regard to parasite intensities, *Strongyloides* had a higher mean in Tana River (136EPG) as opposed to Tsavo being slightly lower (105EPG) with significant difference between the sites (*p* <0.05). *Oesophagostomum* was second still with Tana having a higher mean EPG of 105 compared to Tsavo (99EPG) with no significant difference between the sites (*p* >0.05). *Enterobius* was the next with Tsavo having a higher intensity (88EPG) compared to Tana River (62EPG) with a significant difference between the sites (*p* >0.05). *Trichuris* was the second last in intensity with Tana River being higher (79EPG) as opposed to Tsavo (53EPG) with a significant difference between the sites (*p* <0.05). The lowest in intensity among the helminths was *S. mansoni* which was only reported in Tsavo (50EPG) with a significant difference between the three sites (*p* <0.05) (Figure 3). The intensities were all light intensities when compared to WHO infection intensity scale (WHO, 2002).

## 4 DISCUSSION

Lakipia was reported to have the highest prevalence of helminth infections followed by Tana River then Tsavo. Lakipia being a ranch, there is higher interaction between humans and baboons as compared to the parks. These animals that have moved far away from the parks in search of food move around home and hotels in search of food. In this locality, there are also tourist hotels which serve as an attraction to the baboons since they feed on leftovers and some are fed by tourists. Feeding on leftovers makes them pick humans parasites and, in the process, they also disseminate the parasites through feaces or other discharges (Mafuyai et al., 2013). *Oesophagostomun* had the highest prevalence of all sites. It is however reported that *O. bifucum* strain which infects human is different from the one that infects NHPs (De Gruijter et al., 2005). *Stongyloides* also showed a very high prevalence and was the second highest. These two parasites are still neglected and are not among the current list of NTDs listed by WHO yet they appear very common. Strongyloides is termed as the most neglected among the neglected diseases yet its two species; *S. fuelleborni* and the more prevalent *S. stercoralis* are currently believed to infect an estimated 30–100 million people worldwide yet they are still common parasites of non‐human primates (Howells Michaela et al., 2011). Hopefully, as the other helminths especially soil transmitted helminths are handled through PCT, these helminths will also be cleared. The challenge is that the choice of drug and dose is dependent on the specific parasites and so these were not factored during the decision. Interestingly, *Trichuris* prevalence is quite low, *S. mansoni* which is also known to be focal is also very low in the endemic areas and *Ascaris* was not reported at all in all the three sites. Hospital data from these localities have also gone down drastically due to several rounds of MDAs being carried out by NGOs and ministry of health. This indicates that the elimination of NTDs, especially schistosomiasis and STHs control is in a good course but there are other neglected helminths which will continue causing problems.

Chemotherapy with the conventional drugs showed a very good response. Albendazole is still the drug regularly used for PCT as well as for treatment of most nematodes. This means that with PCT, which in some cases have been done several times especially due to uncoordinated deworming program, most of these parasites are being eliminated in the population. The main challenge remains in the wildlife and especially non‐human primates and more so the baboon being possible sources of new infections due to their interaction with humans (Mafuyai et al., 2013).

Notably all the baboons from all the sites had low level intensities of infection as per the WHO levels (WHO, 2002). In relation to the parasites intensities, *Strongyloides* still was the leading followed by *Oesophagostomum* then *Trichuris* and lastly *Enterobius* with *S. mansoni* being detected only in one site and having very low intensity. The baboons have probably been able to regulate the numbers of these parasites to low level where they don't cause severe clinical manifestation (Egbetade et al., 2014) though in some incidences there were high intensity infections. The low intensities infections have been noted to reduce wildlife population by reduction of their vigor (Dagnachew et al., 2011; McCallum & Dobson, 1995). These animals therefore serve as good reservoirs of the helminths and continue disseminating them in the environment hence continued source of infection to humans.

*Schistosoma mansoni* is reported to be endemic in the eastern region and in Lake Victoria region of the country, while *S. haematobium* has been reported to be mostly endemic in the coastal region but there are now also reports of coendemicity in the Lake Victoria region (Brooker et al., 2009, Sang et al., 2014). Soil transmitted helminths on the other hand are widely distributed in the country (Brooker et al., 2009).

Currently, there is a campaign by WHO to eliminate preventable chemotherapy‐neglected tropical diseases (PCT‐NTDs). The main ones in Kenya are *Trichuris*, *Ascaris,* schistosomiasis, lymphatic filariasis, hookworms and this is done through mass drug administration. The challenge remains that despite elimination from humans, the threat of reinfection still exists from non‐human primates, especially so the baboons whose interaction with humans appears to be the highest among wildlife.

## 5 CONCLUSION

There is a high prevalence of zoonotic helminths in baboons an aspect which have not been dealt with. The control in humans alone will not clear the infections if the baboon infections are not dealt with too as they continue to be reservoirs. This is because human and wildlife interactions are increasing due to increase in human population.

## CONFLICT OF INTEREST

No conflict of interest to declare.

## AUTHOR CONTRIBUTION

**Fredrick Chimoyi Maloba:** Conceptualization; Data curation; Formal analysis; Funding acquisition; Investigation; Methodology; Project administration; Software; Supervision; Validation; Visualization; Writing‐original draft; Writing‐review & editing. **Danson Mwangi:** Conceptualization; Formal analysis; Funding acquisition; Investigation; Methodology; Validation; Visualization; Writing‐original draft; Writing‐review & editing. **John Kagira:** Conceptualization; Formal analysis; Funding acquisition; Investigation; Methodology; Validation; Visualization; Writing‐original draft; Writing‐review & editing. **Sternly Kivai:** Conceptualization; Funding acquisition; Investigation; Methodology; Visualization; Writing‐original draft; Writing‐review & editing. **David Ndeereh:** Conceptualization; Data curation; Investigation; Methodology; Supervision; Validation; Visualization. **Joseph Maina Ngotho:** Conceptualization; Data curation; Formal analysis; Funding acquisition; Investigation; Methodology; Visualization; Writing‐original draft; Writing‐review & editing. **Michael Muita Gicheru:** Data curation; Formal analysis; Investigation; Methodology; Supervision; Validation; Visualization; Writing‐original draft; Writing‐review & editing. **Suleiman Mbaruk:** Data curation; Formal analysis; Investigation; Methodology; Supervision; Validation; Visualization; Writing‐original draft; Writing‐review & editing. **Mercy Akinyi:** Conceptualization; Data curation; Formal analysis; Funding acquisition; Investigation; Methodology; Project administration; Resources; Software; Supervision; Validation; Visualization; Writing‐original draft; Writing‐review & editing.

## ETHICAL STATEMENT

The authors confirm that the ethical policies of the journal, as noted on the journal's author guidelines page, have been adhered to and the appropriate ethical review committee approval has been received. The baboons were humanly treated throughout the study. Prior to carrying out the study, ethical approvals were sort from various institutions. The study was approved by the Institute of Primate Research's Animal Care and Use Committee (ACUC), a sub‐committee of the Institutional Review Committee (approval number IRC/15/11). The research adhered to the legal requirements of the Kenya. The study was also approved by the National Council of Science Technology and Innovation, a body in under the ministry of Science and Technology which approves all scientific research carried out in Kenya (REF NO: STIG/03R/2010‐11). Approval for capturing and sampling of baboons in the wild was also obtained from Kenya Wildlife Service, the government body in charge of wildlife in the Kenya.

### PEER REVIEW

The peer review history for this article is available at https://publons.com/publon/10.1002/vms3.495.

## Data Availability

The data that support the findings of this study are available from the corresponding author upon reasonable request.
